# Common BMI and diabetes-related genetic variants: A pilot study among indigenous people in the Brazilian Amazon

**DOI:** 10.1590/1678-4685-GMB-2021-0153

**Published:** 2022-05-11

**Authors:** Isabela Guerreiro Diniz, Rosilene Reis Della Noce, Ana Paula Pereira, Aylla Núbia Lima Martins da Silva, Eliene Rodrigues Putira Sacuena, Renan Barbosa Lemes, Greice de Lemos Cardoso-Costa, Gilderlânio Santana Araújo, Jéssica Lígia Picanço Machado, Fernanda Andreza de Pinho Lott Figueiredo, Tábita Hümemeier, João Farias Guerreiro

**Affiliations:** 1 Universidade Federal do Pará, Instituto de Ciências Biológicas, Laboratório de Genética Humana e Médica, Belém, PA, Brazil.; 2 Universidade Federal do Pará, Instituto de Ciências da Saúde, Faculdade de Nutrição, Belém, PA, Brazil.; 3 Universidade de São Paulo, Departamento de Genética e Biologia Evolutiva, São Paulo, SP, Brazil.

**Keywords:** T2D, BMI, SNP, Amerindians, Brazilian

## Abstract

This study was carried out to investigate the frequency of genetic variants related to body mass index (BMI) and type 2 diabetes (T2D) and evaluating the potential impact of risk alleles on susceptibility to these disorders in six indigenous peoples from Brazilian Amazon region. The majority of Fst values for pairwise population comparisons among the indigenous groups are low or moderate. The indigenous people show high values of differentiation with Africans, Europeans and Southeast Asians and moderate values with East Asian and American populations, as expected. The allelic frequencies among indigenous indicate that the majority of associations observed with T2D in continental populations can be replicated in native Amazonians. The genetic risk scores calculated for T2D in indigenous are high and similar to those calculated for Americans and East Asians, while the estimates obtained for obesity are low, probably due to the low frequencies of the risk allele of the *FTO* gene found in our samples. *ADRB3*-rs4994 and *ABCC8*-rs1799854 genes showed a significant association with BMI and waist circumference, and the *KCNJ11*-rs5219 gene with hyperglycemia. These results emphasize the importance of knowing the genetic variability underlying complex genetic diseases in indigenous peoples and the search for particular or rare variants.

## Introduction

Obesity and T2D are complex genetic disorders caused by a combination of genetic, epigenetic and environmental factors. They are related disorders, since obesity is a strong risk factor for T2D, and both conditions are strongly hereditary. Family-based studies clearly show that genetic factors play an important role in the susceptibility to T2D. More than 400 genetic risk variants in 250 loci for T2D have been identified, mainly through broad genome association studies (GWASs) (Dziewulska et al., 2018). With regard to obesity, evidence from family and twin studies has shown that approximately 40-70% of the variation in obesity in humans is attributed to genetic factors (Sandholt et al., 2012), and at least 551 genetic loci associated with obesity were identified by GWAS (Young et al., 2018). Recently, approximately 54 risk alleles for obesity and BMI (OR> 1 and p-value ≤ 10-8) were found using the Disease-Ancestry Network (DANCE) tool, a graph-based web tool that allows integration and visualization of information related to complex human phenotypes and their hits in GWAS, as well as their frequencies of risk alleles in different populations (Araújo et al., 2016).

Epidemiological data have revealed that certain racial and ethnic groups are more (or less) likely to develop obesity and T2D. These data indicate a higher prevalence of obesity, T2D and dyslipidaemia in Native Americans, as well as in “Latinos” or “Mestizos” (resulting from the mixture between Native Americans and Europeans), than in Euro-Americans and that the risk for these pathologies increases with the highest proportion of indigenous ancestry (Avilés-Santa et al., 2017; Young et al., 2018; Morales et al., 2020). In this sense, a study using a polygenic risk score found that T2D genetic risk in Colombian and Hispanic populations from the United States is positively correlated with African and Native American ancestry and negatively correlated with European ancestry, supporting the previous epidemiological data (Chande et al., 2020). Nevertheless, for both T2D and obesity, most of the genetic data available result from studies conducted predominantly in European populations and in Asians on a smaller scale, while Latin populations and Native American populations are rarely studied (Dziewulska et al., 2018; Young *et al.*, 2018). 

Brazilian indigenous populations are potential targets for the studies of complex traits, such as obesity, since indigenous people are influenced by external factors in a more homogeneous way, which allows for more efficient identification of rare and low-frequency genetic variants. In this context, this pilot study was carried out in indigenous populations in which the degree of admixture with non-indigenous people is still low, which allows minimal influence of genes from miscegenation with other ethnicities. The aims were to analyse the prevalence of some variants of common risks associated with obesity and comorbidities in continental populations, to describe the genetic profile of indigenous populations in relation to these variants and to assess the potential impact of this genetic variability on susceptibility to these disorders.

## Subjects and Methods

### Study population

Arara: Karib-speaking individuals who live in six villages located near the Laranjal stream in the municipality of Altamira, PA, with a population of 349 individuals, and in four villages located near the Cachoeira Seca stream on the upper Iriri river, with 252 indigenous people (-3.71603, -53.0546).

Araweté: The Araweté, of Tupi-Guarani language, currently inhabit 12 villages on the banks of the Xingú River and in the Ipixuna igarapé, a tributary of the right bank of the Middle Xingu, in the municipality of Altamira, PA. Population, 559 (-4.88533, -52.42809).

Asurini do Xingú: Located in Pará, municipality of Altamira close to Igarapé Ipixuna, they have 260 individuals currently distributed in five villages on the banks of the Xingú River. Language of the Tupi-Guarani family (-4.24488, -52.23805).

Gavião Kyikatêjê: Located in the Mãe Maria Indigenous Land in the municipality of Bom Jesus do Tocantins, southeastern Pará. Language of the Jê family, with a current population of about 400 inhabitants (-5.10796, -48.9180).

Parakanã: Located in the Xingu basin in the municipalities of São Félix do Xingu, Pará. Language of the Tupi-Guarani family, with a population of 716 people. They currently live in 11 villages (-5.6904, -52.0037).

Xikrin do Bacajá: At present, they live in 11 villages located on the banks of the middle Bacajá River, in the municipalities of Senador José Porfírio and Anapú, PA. Kayapó language (or Mebengokré), from the Jê language family. Current population, 1127 (-3.71603, -53.0546).

**Figure 1 -:**
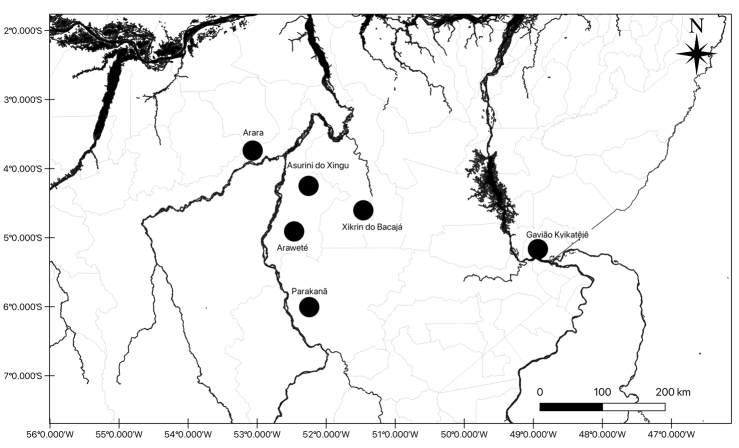
Geographical location of the studied indigenous peoples in the state of Pará, Brazilian Amazon.

The above information is presented in Table 1 and the locations of indigenous lands are shown in Figure 1.

**Table 1-:** Number of individuals, population locality and linguistic group of the studied Amazonian populations.

Population	Number of individuals	Locality	Geographic coordinates	Linguistic group
Arara	601	Altamira, PA	(-3.71603, -53.0546)	Karib
Araweté	559	Altamira, PA	(-4.88533, -52.42809)	Tupi-Guarani
Asurini do Xingú	260	Altamira, PA	(-4.24488, -52.23805)	Tupi-Guarani
Gavião Kyikatêjê	400	Bom Jesus do Tocantins, PA	(-5.10796, -48.9180)	Jê
Parakanã	716	São Félix do Xingu, PA	(-5.6904, -52.0037)	Tupi-Guarani
Xikrin do Bacajá	1127	Senador José Porfírio and Anapú, PA	(-3.71603, -53.0546)	Jê

This was a pilot study of genetic epidemiology carried out with five indigenous peoples located in the southwest of Pará (Arara, Araweté, Asurini do Xingu , Parakanã, Xikrin do Bacajá) and the Gavião Kyikatêjê people, in southeastern Pará, Brazilian Amazon. The epidemiological study was carried out with 628 indigenous people (332 women and 296 men) aged 18 or over, of both sexes, during healthcare visits carried out between 2007 and 2014, in cooperation with health teams from the National Health Foundation (Funasa) and later from the Special Indigenous Sanitary District (DSEI), of the Ministry of Health. Obtaining clinical and biological data was preceded by a meeting with the community to explain the work, benefits and risks, procedures used, and to obtain the communities’ consent through its leaders with formal collective authorization. All residents of the villages were invited to participate in the service and were instructed on the need for fasting for biochemical tests. All participants were evaluated in a medical consultation, starting the treatment of conditions that could be confirmed in the field. The adhesion of indigenous communities to health actions was high and the percentage of participants among those eligible was 55% among the Araweté, above 70% among the Asurini do Xingu, Parakanã and Gavião Kyikatêjê, and 100% among the Arara.

After 9 hours of fasting, blood samples (4 mL) were collected into vacuum collection tubes containing sodium fluoride as a glycolytic inhibitor and the EDTA anticoagulant to determine blood glucose levels, and 4 ml were collected in tubes without additive to determine total cholesterol and triglycerides. These measurements were performed in the field laboratory. The fasting plasma glucose (FPG) results were classified as follows: normoglycemia, <99 mg/dL; impaired fasting glucose (IFG), 100-125 mg/dL; diabetes, ≥125 mg/dL. Dyslipidemia was defined as a serum cholesterol level ≥ 200 mg/dL or triglyceride level ≥150 mg/dL.

Hypertension was defined as a systolic blood pressure (SBP) ≥ 140 mm/Hg and/or a diastolic blood pressure (DBP) ≥ 90 mm/Hg. Body mass index (BMI) classification used the WHO criteria for adults (WHO, 2000). A waist circumference (WC) < 90 cm in men and < 80 cm in women was considered normal (International Diabetes Federation, 2006).

### SNP genotyping

Genomic DNA was extracted from 300 mL of EDTA-treated blood using the NeoIsoColumn kit (One Lambda Inc., San Diego, CA, USA) according to the manufacturer’s instructions. DNA was eluted into 200 μL of elution buffer (provided with the kit). The following single nucleotide polymorphisms (SNPs) were analysed: *CAPN10*-rs3792267, *CAPN10*-rs5030952, *ABCC8*-rs1799854, *KCNJ11*-rs5219 *CTF7L2*-rs7901695, *CTF7L2*-rs12255372 and *PPARG*-rs1801282, associated with T2D. *ADRB3*-rs4994 and *FTO*-rs8050136, associated with obesity; and *ABCA1*-rs9282541, associated with low levels of high-density lipoprotein (HDL-C). Genotyping was done using a TaqMan SNP genotyping assay (Applied BioSystems, Foster City, CA, USA) according to the manufacturer’s instructions. Pre-designed probes were ordered for genotyping analyses. Approximately 10-50 ng of DNA was amplified with 5 μl of 2X TaqMan Universal PCR master mix, 0.5 μl of 40X primer and TaqMan probe dye mix. Cycling conditions consisted of 10 min at 95 °C, followed by 40 cycles of 15 s at 92 °C and 1 min at 60 °C. Allelic discrimination was performed on an Applied BioSystem RT-PCR system - Real-time PCR. These genes were selected for this pilot study using as a criterion previous studies demonstrating the association of variants with obesity and/or type 2 diabetes in continental populations, and for which there were results described for Native American people, from Brazil, in particular, or for the Brazilian population in general.

### Statistical analysis

Allele frequencies, Hardy-Weinberg equilibrium (HWE) for each locus, mean heterozygosities, interpopulation variability (Fst) and analysis of molecular variance (AMOVA) were determined using the Arlequin v.3.5 program (Excoffier and Lischer, 2010). Effective population sizes (Ne) for each polymorphism were estimated using the formula Ne=N/(1+f), where N is the sample size and f is the inbreeding coefficient. The biological variables were compared using Student’s t test or the U test of Mann-Whitney for continuous variables and Pearson’s χ2 test for categorical variables. Logistic regression under *a priori* genotypic models (dominant, co-dominant and recessive) was performed to calculate risk allele-specific odds ratios (ORs), 95% confidential intervals, and corresponding p-values, after adjusting for sex and age. These analyses were performed with the Statistical Package for the Social Sciences (SPSS) for Windows, version 20.0 (SPSS Inc., Chicago, IL, USA). An overall significance level of 0.05 was set for statistical analyses, considering that the tests were applied to 10 genetic markers, the critical p-value to be considered after Bonferroni correction is 0.005. Composite genetic scores (CGS) for obesity and type 2 diabetes, considering the cumulative effect of all variants on the basis of the number of risk SNPs and the number of copies of risk alleles, were estimated at the population level according to Mao et al. (2017). Typical confounding effects are probably negligible, since no genotyping or sample ascertainment bias are expected, and we found no clear population structure (see section Results).

## Ethical aspects

This study was approved by the National Research Ethics Committee (CONEP) Report no. 1,062/2.006 and Report no. 961,451/2,015).

## Results

### Anthropometric and metabolic variables

Overall, 628 individuals were studied (52.9% women and 47.1% men), with ages ranging from 18 to 88 years and from six indigenous groups in the Brazilian Amazon: Arara, Araweté, Asurini do Xingú, Parakanã, Gavião Kyikatêjê and Xikrin do Bacajá. The mean age was 38.7 ± 16.6 years, and there was no difference between men (39.6 ± 17.3 years) and women (37.8 ± 15.9 years) (p = 0.089). 

Anthropometric and metabolic variables are presented by sex in Table 2. The overall prevalence of excess weight among indigenous groups was 33.4% (180/538); 26.0% (140/538) were classified as being overweight, and 7.4% (40/538) were diagnosed as obese. There were no statistically significant differences in the prevalence of overweight and obesity between men and women. However, central obesity, found in 39.5% (203/514) of indigenous adults, was more prevalent in women (WC: 62.5% vs 13.6%) with p = 0.00, OR (95% CI) = 0.09 (0.06 - 0.15). The highest proportions of obesity were observed in the Asurini do Xingú (24.3%) and Gavião Kyikatêjê (26.8%) peoples, with similar frequencies in both sexes. A high prevalence of abdominal obesity was also found in the Asurini do Xingú (70.3%) and Gavião Kyikatêjê (72.0%), with the prevalence being significantly higher in women: 90.9% vs 40.0%, p = 0.00, OR (95%CI) = 0.05 (0.01 - 0.35) among Asurini do Xingú, and 93.8% 3 vs 33.3%, p = 0.00, OR (95%CI) = 0.03 (0.01 - 0.19) among the Gavião Kyikatêjê.

**Table 2 -:** Nutritional status and metabolic profile of six indigenous peoples in the Brazilian Amazon according to sex.

Variable	Arara		Araweté		Asurini		Xikrin		Parakanã		Gavião		Overall		
	Female	Male	Female	Male	Female	Male	Female	Male	Female	Male	Female	Male	Female	Male	Total
IFG															
N	5	2	3	2	4	3	11	8	0	3	3	1	26	19	45
%	10.2	4.1	8.6	5	18.2	20	11	8.7	0	5.2	7.1	2.8	8.2	6.6	7,4%
Diabetes															
N					1	0	1	0	0	1	4	0	6	1	7
%					4.5	0	1	0	0	1.7	9.5	0	1.9	0.3	1,2%
Dyslipidemia															
N	14	6	14	6	11	9	29	23	17	12	15	14	93	74	167
%	29.2	12.5	29.2	12.5	61.1	64.3	31.2	27.7	24.6	24	35.7	38.9	31.5	29	30,4%
Overweight															
N	11	15	5	1	4	5	25	30	19	8	10	7	74	66	140
%	25.6	31.9	16.7	2.9	18.2	33.3	28.7	37	29.2	14	28.6	33.3	26.2	25.8	26,0%
Obesity															
N	1	1			6	3	8	2	2	2	10	5	27	13	40
%	2.3	2.1			27.3	20	9.2	2.5	3.1	3.5	28.6	23.8	9.6	5.1	7,4%
Elevate WC															
N	17	1	11	0	20	6	56	16	36	4	30	6	170	33	203
%	48.6	2.9	36.7	0	90.9	40	66.7	19.5	52.2	6.9	93.8	33.3	62.5	13.6	39.5
Hypertension															
N	0	1	1	0	0	1	0	4	0	1			1	7	8
%	0	2.1	3.1	0	0	6.7	0	4.5	0	1.8			0.4	2.9	1,6%

IFG: Impaired Fasting Glucose.

High levels of fasting blood glucose were found in 8.6% of the investigated sample (52/607), of which 1.2% were diagnosed as type 2 diabetes. Men and women had similar prevalence.

The overall prevalence of dyslipidemia was 30.4% (women, 31.5%; men, 29.0%), and the highest proportion was observed among the Asurini do Xingú (30.0%). The prevalence of arterial hypertension was 1.8% in total, and there was no difference between genders.

### Genotype and allele frequencies

The allele frequencies observed for 10 variants in eight genes related to T2D and obesity in the six indigenous populations are shown in Table 3, together with the frequencies found in continental populations of the “1000 Genomes Project” (Africans, Americans, Europeans, Southeast Asians and East Asians). The American subset of the 1000 Genomes Project is composed by admixed populations from Puerto Rico, Mexico, Colombia, and Peru.

**Table 3-:** Allele frequencies for 10 single-nucleotide polymorphisms (SNPs) of eight genes related to obesity and diabetes in six Amerindian groups and in continental populations from 1000 Genomes Project (in %).

Gene/SNP	Allele	Population											
		ARA	ARW	ASX	XKB	PRK	GAV	BRA	EUR	AFR	AMR	EAS	SAS
CAPN1043	G	50.5	96.0	45.9	59.8	61.6	65.3	63.2	72.9	84.4	71.9	92.4	82.1
rs3792267	A	48.5	4.0	54.1	40.2	38.4	34.7	36.8	27.1	15.6	28.1	7.6	17.9
CAPN1063	C	49.5	46.0	66.2	52.8	41.9	51.7	49.7	92.5	46.2	79.7	75.8	93.8
rs5030952	T	50.5	54.0	33.8	47.2	58.1	48.3	50.3	7.5	53.8	20.3	24.2	6.2
ABCC8	G	69.4	76.7	50.0	29.3	37.6	41.5	46.7	58.0	87,5	46.4	44.9	68.1
rs1799854	A	30.6	23.3	50.0	70.7	62.4	58.5	53.3	42.0	12.5	53.6	55.1	31.9
KCNJ11	C	88.8	46.7	58.1	66.2	40.7	49.2	59.5	64.7	99.7	70.7	66.2	60.4
rs5129	T	11.2	53.3	41.9	33.8	59.3	50.8	40.5	35.3	0.3	29.3	33.8	39.6
CTF7L2A	C	3.1	0.7	0.0	16.2	2.7	13.6	7.6	34.4	42.9	25.6	2.3	29.6
rs7901695	T	96.9	99.3	100.0	83.8	97.3	86.4	92.4	65.6	57.1	74.4	97.7	70.5
CTF7L2B	G	99.5	100.0	98.6	99.7	99.2	97.5	99.3	70.8	68.5	78.1	99.0	77.9
rs12255372	T	0.5	0.0	1.4	0.3	0.8	2.5	0.7	29.2	31.5	21.9	1.0	22.1
PPARG	C	89.8	88.7	79.7	73.5	74.4	66.9	78.2	88.0	99.9	88.3	97.4	88.0
rs1801282	G	10.2	11.3	20.3	26.5	25.6	33.1	21.8	12.0	0.1	11.7	2.6	12.0
ADRB3	A	67.8	82.7	48.6	58.9	37.6	67.8	60.7	91.8	90.2	88.0	86.9	84.3
rs4994	G	32.2	17.3	51.4	41.1	62.4	32.2	39.3	8.2	9.8	12.0	13.1	15.7
FTO	C	100.0	100.0	95.9	85.8	100.0	97.5	95.1	58.6	57.1	74.5	83.4	71.1
rs8050136	A	0.0	0.0	4.1	14.2	0.0	2.5	4.9	41.4	42.9	25.5	16.6	28.9
ABCA1	G	89.3	78.0	95.9	83.0	99.6	95.8	89.3	100.0	1.00	95.8	100.0	100.0
rs9282541	A	10.7	22.0	4.1	17.0	0.4	4.2	10.7	0.0	0.0	4.2	0.0	0.0

ARL, Arara; ARW, Araweté; ASX, Asurini do Xingu; XKB, Xikrin do Bacajá; PRK, Parakanã; GAV, Gavião Kyikatejê; BRA, Brazilian Amerindians; EUR, European AFR, African; AMR, American; EAS, East Asian; SAS, Southeast Asian. 

The intrapopulation variability observed in indigenous populations based on the expected average heterozygosity under the Hardy-Weinberg hypothesis for all loci in the set of indigenous populations was 0.320 ± 0.183, ranging from 22.9% in Araweté to 35.3% in Xikrin do Bacajá (Table 4). Hardy-Weinberg disequilibrium was observed in loci *ABCA1* (rs9282541) in the Araweté people, *FTO* (rs8050136) in Gavião Kyikatêjê, and *CAPN1063* (rs5030952) and *CTF7L2B* (rs12255372) in Parakanã. The effective population sizes Ne obtained for all polymorphisms studies were presented in Table 5, the mean value for all polymorphisms is 95.5 individuals, ranging from 33.79 to 212.22.

**Table 4 -:** Population diversity parameters among Brazilian Amerindians and continental populations of 1000 Genomes Project.

Population	Genotypes	Obs. Het	Exp Het	Loci HWD	Mono. loci
Xikrin do Bacajá	179	0.347	0.353	0	0
Gavião Kyikatêjê	59	0.300	0.326	1	0
Arara	98	0.274	0.269	0	1
Araweté	75	0.285	0.286	1	2
Asurini do Xingú	37	0.351	0.331	0	1
Parakanã	129	0.293	0.285	2	1
African	505	0.290	0.286	1	0
American	347	0.319	0.324	0	0
East Asian	504	0.228	0.230	0	1
European	503	0.348	0.354	1	1
Southeast Asian	489	0.324	0.320	0	1

Observed Het = Average Observed Heterozygosity; Expected Het = Average Expected Heterozygosity; HWD = Hardy-Weinberg Disequilibirum; Mono. Loci = Monomorphic loci.

**Table 5-:** Effective population sizes of Brazilian Amazonian populations obtained from 10 genetic markers.

SNP	Populations					
	Arara	Araweté	Asurini Xingú	Gavião Kyikatejê	Parakanã	Xikrin Bacajá
rs3792267	106.8291	78.2609	40.5806	50.2206	174.8097	181.7534
rs5030952	106.7242	70.711	38.0243	46.1558	163.3677	160.9387
rs1801282	80.298	73.7681	34.2521	57.611	125.4784	212.218
rs4994	110.5641	94.898	40.2994	47.4059	114.7263	161.2665
rs9282541	111.3636	103.7805	38.6324	62.7273	129.5039	160.3604
rs7901695	101.1957	75.5068	-	47.3103	101.9123	169.0546
rs12255372	98.5052	-	37.5139	57.5849	64.5	184.5027
rs5219	100.4133	72.4138	51.1629	56.0716	130.5357	175.7143
rs1799854	86.3212	61.6071	38.0278	50.2411	118.1995	168.9462
rs8050136	-	-	38.6324	33.786	-	180.6016

The levels of genetic differentiation observed among indigenous groups based on Fst values were classified as low (≤ 0.05) or moderate (> 0.05 and ≤ 0.15) in most of the paired comparisons. However, the Araweté exhibited somewhat higher differentiation coefficients (0.168) in relation to the Asurini do Xingú and Parakanã people, which in turn also exhibited a somewhat higher coefficient in relation to the Arara. Regarding the continental populations, the indigenous people presented high values of differentiation in comparison with Africans, Europeans and Southeast Asians, in decreasing order. Fst values indicative of moderate genetic differentiation (<0.15) were observed in relation to East Asian and American populations (Table 6).

**Table 6 -:** Population pairwise FSTs values for Brazilian Amazon indigenous groups and continental populations from 1000 Genomes Project.

	AFR	AME	EAS	EUR	SAS	XKB	GAV	ARA	ARW	ASK	PRK
AFR	0.000										
AME	0.139	0.000									
EAS	0.235	0.059	0.000								
EUR	0.137	0.022	0.113	0.000							
SAS	0.147	0.028	0.087	0.015	0.000						
XIK	0.253	0.083	0.129	0.155	0.154	0.000					
GAV	0.271	0.086	0.126	0.153	0.138	0.020	0.000				
ARA	0.196	0.112	0.156	0.181	0.167	0.090	0.103	0.000			
ARW	0.245	0.144	0.127	0.197	0.155	0.139	0.105	0.139	0.000		
ASK	0.301	0.109	0.162	0.172	0.155	0.036	0.034	0.085	0.168	0.000	
PRK	0.351	0.178	0.212	0.247	0.229	0.054	0.036	0.166	0.168	0.041	0.000

ARA, Arara; ARW, Araweté; ASX, Asurini do Xingu; XKB, Xikrin do Bacajá; PRK, Parakanã; GAV, Gavião Kyikatejê; BRA, Brazilian Amerindians; EUR, European AFR, African; AMR, American; EAS, East Asian;; SAS, South Asian. Fst = <0.05: low genetic differentiation; Fst = 0.05-0.15: moderate genetic differentiation; Fst => 0.15 high genetic differentiation;

The analysis of molecular variance (AMOVA) revealed that most of the genetic variation among indigenous people occurs within populations (~90%) and that ~10% occurs among populations. When the AMOVA includes samples from the 26 populations of 1000 Genomes Project grouped according to their continental regions and our six populations grouped in an Amazonian cluster, most of the genetic variation also occurs within populations (85.5%), while the variation that occurs among groups is 12% and the variation among populations within groups is 2.5%.

### Genotype-phenotype association

Although the general prevalence of metabolic (blood glucose, cholesterolemia and triglyceridemia) and anthropometric (BMI and waist circumference) changes in the indigenous populations studied are still low to allow robust analyzes of the association with genetic variants, the *ADRB3* (rs4994) gene showed a significant association with waist circumference measurements and BMI. The *ABCC8* (rs1799854) gene and the *KCNJ11* gene (rs5219) showed lower p-values (<0.05) in association analysis with BMI and hyperglycemia, respectively, under dominant and co-dominant models (Table 7), although not statistically significant when Bonferroni correction is considered.

**Table 7 -:** Odds ratio adjusted for three genetic models for common variants associated with anthropometric changes and hyperglycemia.

Gene	Genotype	Normal WC	Elevated WC	Co-Dominant OR (95%CI) (GG vs GA, AA	Dominant OR (95%CI) (GG vs GA+AA	Recessive OR (95%CI) (GG+GA vs AA
ABCC8	GG	90 (29.7%)	36 (19.8%)	1.0	1.0	1.0
rs1799854	GA	118 (38.9%)	78 (42.9%)	1.63 (1.01 - 2.64); p=0.04	1.68 (1.08 - 2.62); p =0.02	1.28 (0.87 - 1.89); p = 0.21
	AA	95 (31.4%)	68 (37.4%)	1.74 (1.06 - 2.88); p = 0.03		
Gene	Genotype	Normal WC	Elevated WC	Co-Dominant OR (95%CI) (AA vs AG, GG	Dominant OR (95%CI) (AA vs AG+GG	Recessive OR (95%CI) (AA+AG vs GG
ADRB3	AA	133 (43.9%)	54 (29.7%)	1.0	1.0	1.0
rs4994	AG	118 (38.9%)	84 (46.2%)	1.75 (1.14 - 2.67); p = 0.01	1.84 (1.24 - 2.72); p = 0.00	1.52 (0.96 - 2.39); p = 0.07
	GG	52 (17.2%)	44 (24.2%)	2.06 (1.23 - 3.43); p = 0.00		
Gene	Genotype	Normal	Excess weight	Co-Dominant OR (95%CI) (AA vs AG, GG	Dominant OR (95%CI) (AA vs AG+GG	Recessive OR (95%CI) (AA+AG vs GG
ADRB3	AA	148 (43.4%)	54 (32.3%)	1.0	1.0	1.0
rs4994	AG	132 (38.7%)	79 (47.3%)	1.63 (1.07 - 2.48); p = 0.02	1.58 - (1.07 - 2.34); p = 0.02	1.15 (0.72 - 1.84); p = 0.56
	GG	61 (17.9%)	34 (20.4%)	1.49 (0.88 - 2.52); p = 0.14		
Gene	Genotype	Normal	Hyperglycemia	Co-Dominant OR (95%CI) (TT vs TC, CC	Dominant OR (95%CI) (TT vs TC+CC	Recessive OR (95%CI) (TT+TC vs CC
KCNJ11	TT	108 (20.1%)	3 (6.2%)	1.0	1.0	1.0
rs5219	TC	228 (42.5%)	25 (52.1%)	3.83 (1.13 - 12.9); p = 0.03	3.79 (1.15 - 12.4); p = 0.03	0.28 (0.70 - 2,37); p = 042
	CC	201 (37.4%)	20 (41.7%)	3.75 (1.09 - 12.9); p = 0.03		

### Genetic risk scores

**Figure 2 -:**
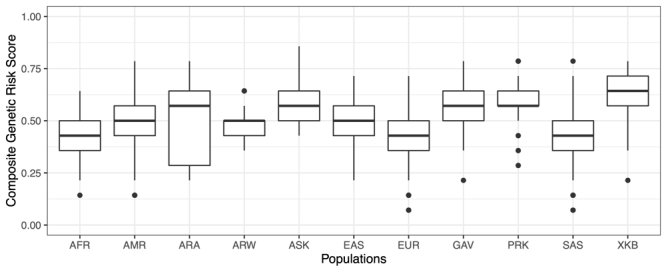
Genetic risk scores for diabetes in indigenous and continental populations. ARA, Arara; ARW, Araweté; ASK, Asurini do Xingu; XKB, Xikrin do Bacajá; PRK, Parakanã; GAV, Gavião Kyikatêjê; EUR, European AFR, African; AMR, American; EAS, East Asian; SAS, Southeast Asian.

**Figure 3-:**
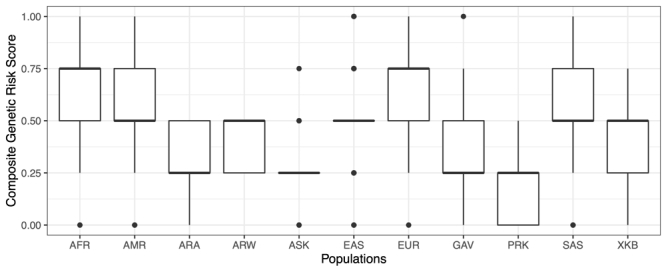
Genetic risk scores for BMI in indigenous and continental populations. ARA, Arara; ARW, Araweté; ASK, Asurini do Xingu; XKB, Xikrin do Bacajá; PRK, Parakanã; GAV, Gavião Kyikatêjê; EUR, European AFR, African; AMR, American; EAS, East Asian; SAS, Southeast Asian.

The estimated cumulative effect of all variants associated with T2D (*CAPN1043*, *CAPN1063*, *ABCC8*, *KCNJ11*, *CTF7L2A*, *CTF7L2B* and *PPARG*), revealed mean risk scores for ranging from 0.484 in the Arara group to 0.620 in the Xikrin do Bacajá group. For obesity (*ADRB3* and *FTO* genes) the lowest average risk score was calculated for the Parakanã (0.188) and the highest for the Araweté group (0.413) (Figures 2 and 3).

## Discussion

Overall, the prevalence of excess weight (31.7%) among the natives of the Brazilian Amazon analysed in this study was lower than that described for urban Brazilian adults (63.1%), as was the prevalence of obesity (6.4% vs 22.9%) (Schmidt *el al* ., 2015) and central obesity (37.4% vs 80.7%) (Cardinal et al., 2018). However, the analysis by indigenous people revealed a high prevalence of obesity and abdominal obesity in the Gavião Kyikatêjê (26.7% and 76.0, respectively) and Asurini do Xingú (24.0% and 70.3%, respectively), which are similar to those described for non-indigenous Brazilian adults.

The prevalence of metabolic changes among the Amazonian indigenous peoples studied here (IFG, 7.5%, T2D, 0.9%) was still very low, being much lower than that observed in non-indigenous Brazilian adults: 52.6% IFG and 20.0% T2D (Schmidt et al., 2015). They are similar to those observed in most of the indigenous groups already studied, but lower than those found in the Xavante people (IFG, 32.3%, T2D, 28.3%). (Dal Fabbro et al., 2014). The prevalence of dyslipidemia (30.1%) was also lower than that estimated for non-indigenous Brazilian adults (61.5%) (Schmidt *et al.*, 2015), similar to that found in most Brazilian Amerindian groups, but lower than that found in some indigenous peoples, such as the Xavante, Aruák, Kaingang-Guarani, Gavião Parkatejê, Suyá and Khisêdjê, in which the prevalences were above 30.0% (Baldoni et al., 2019).

The whole indigenous population investigated here exhibited low prevalence of nutritional and metabolic changes, with the exception of the Gavião Kyikatêjê and Asurini do Xingu, in which high frequencies of obesity and abdominal obesity were observed, certainly as a reflection of accentuated cultural and environmental changes experienced by these groups. The available data reveal that the process of “westernization” of customs is ongoing, to varying degrees, and that the nutritional and epidemiological transitions are at early stages. 

### Allele frequencies in indigenous and continental populations

Although the majority of genetic variants associated with obesity and T2D have been identified from studies carried out predominantly in European populations, and in Asians and Africans to a lesser extent, data have shown that allelic associations of a significant majority of these variants are replicated in populations from other continents (Carlson et al., 2013). The comparison of the frequencies of risk alleles in the indigenous peoples studied here with the frequencies of the populations of the 1000 Genomes Project reveals that in six (CAPN1043 - rs3792267, CAPN1063 - rs5030952, ABCC8 - rs1799854, KCNJ11 - rs5129, CTF7L2A - rs7901695 and PPARG - rs1801282) of the seven analysed variants related to T2D the frequencies are as high in indigenous people as in continental groups. Therefore, it is possible that significant associations can be obtained with these risk alleles in indigenous populations (Table 2). Regarding the variants related to obesity, the risk allele (G) of the rs4994 variant (*ADRB3*) was found with slightly higher frequencies (average of 39.3%) in indigenous people than in continental populations (frequencies ranging from 8.2% in Europeans to 15.7% in Southeast Asians) and can be considered a good marker for association studies in indigenous. However, in the *FTO* gene, the risk allele (A) of the rs8050136 variant was absent or found at very low frequencies among indigenous people. So, we could infer that this estimate of genetic risk was weak because of the low allelic frequency found for FTO in associations with changes in BMI in indigenous people of the Amazon. *FTO* has been recognized as a major genetic factor for obesity risk in different human groups. In addition to showing signs of balancing selection in Europeans (Liu et al., 2015), this gene also has different risk scores across populations, possibly due to different patterns of linkage disequilibrium among continental populations (Adeyemo et al., 2010). In the Brazilian admixed population, the *FTO* polymorphisms were associated with the development of obesity in adults (Ramos et al., 2012; Fonseca et al., 2020) and in children and youth (da Silva et al., 2013; Reuter et al., 2016), probably due to the high percentage of European ancestry in the studied samples. Considering that, our findings deserve further studies in other indigenous peoples and, including more *FTO* variants to identify variants that may be associated with anthropometric changes in these peoples. 

Finally, the risk allele (A) of rs9282541 (*ABCA1*), found with frequencies ranging from 0.4% to 22% (average of 10.7%) in natives of the Amazon, was found only in Americans (4.2%) in the 1000 Genomes Project. This variant of the *ABCA1* cholesterol transporter gene (ATP-binding cassette transporter A1) is associated with low levels of high-density lipoprotein (HDL-C), constituting a functional variant that may have contributed to the adaptive evolution of Native American populations (Acuña-Alonzo et al., 2010; Hünemeier et al., 2012). Apparently, it is exclusive to Native Americans, and the identification of a heterozygous G/A individual in a sample with African ancestry from the southwestern USA, included in the 1000 Genomes Project database, is certainly due to admixture with Native Americans. As the *ABCA1* gene is also involved in the internalization of pathogens, in addition to its role in cholesterol metabolism, the frequency of the derived allele could be influenced by the high prevalence of pathogens in the tropical region (Hünemeier *et al.*, 2012). However, there is no difference between the allele distribution pattern in the populations studied here and those genotyped in previous works (Acuña-Alonzo *et al.*, 2010; Hünemeier *et al.*, 2012). Thus, the distribution of the allele in America appears to be more homogeneous in agricultural populations from Mexico, and with variable distribution in South America, corroborating previously published results (Hünemeier *et al.*, 2012).

The analysis of the distribution of risk alleles also showed that, for the set of genes studied, indigenous populations had high genetic differentiation (Fst) compared to Africans, Europeans and Southeast Asians, but a genetic differentiation classified as moderate in relation to American populations and East Asian populations. This finding is in line with expectations considering that available data confirm that current Native Americans are descended from human expansions that entered North America from East Asia. However, issues such as number, mode and time of secondary migrations to the Americas are still the subject of intense debate (Skoglund and Reich, 2016; Castro e Silva *et al.*, 2021).

### Association between risk alleles and anthropometric and metabolic variables

The β3 adrenergic receptor, encoded by the *ADRB3* gene, is an important regulator of several physiological functions, such as thermogenesis in brown adipose tissue, lipolysis in white adipose tissue, negative inotropic effects on cardiomyocytes and relaxation of blood vessels. Epidemiological studies in different populations have identified polymorphic variants associated with obesity, T2D, cardiovascular diseases and other disorders (Masuo and Lambert, 2011; Yang and Tao, 2019). Thus, the association found between the risk allele (G) of the rs4994 (*ADRB3*), the most commonly investigated *ADRB3* variant, with abdominal obesity and excess weight among indigenous people corroborates results already described in different populations. The *ABCC8* and *KCNJ11* genes are responsible for the coding of ATP-dependent potassium channels (KATP) expressed in pancreatic beta-cells, which play a central role in the glucose-stimulated insulin secretion of pancreatic beta cells. These two genes (*ABCC8* and *KCNJ11*) are adjacent and located on chromosome 11p15.1. Studies have shown that variants in *KCNJ11* are associated with T2D in Europeans in the United Kingdom (Gloyn et al., 2003), and variants in *ABCC8* have been correlated with T2D in Japanese populations (Sakamoto et al., 2007), Caucasians (Florez et al., 2004) and Chinese (Zhou et al., 2019). Thus, the low p-values (<0.05) from association of the *ABCC8* gene (rs1799854) with abdominal obesity found among the indigenous people studied here is probably a spurious association and unrelated to biological processes. The low p-values (<0.05) from association of *KCNJ11* (rs5219) with hyperglycemia, on the other hand, is in line with that observed in other populations.

Although our samples are representative of the populations studied and the care taken with regard to phenotypic data collection, our study has some intrinsic limitations, especially with regard to sample size and to complex traits. Association studies are more robust when applied to large samples (a few thousand individuals), especially when considering the application of correction of multiple tests (such as Bonferroni), something difficult to be circumvented when considering the small sizes of the Amazonian isolates. Also, it is difficult to find association of genetic markers with complex traits, as multiple genetic factors contribute to the phenotype. Additional studies in Amazonian populations may give us more clues about variants related to the important phenotypes considered in this study.

### Genetic risk scores

The comparison of the average genetic risk scores estimated for T2D in indigenous peoples and continental populations showed that the values observed in indigenous (0.592) are high and more similar to those calculated for East Asians (0.503) and Americans (0.481), and slightly higher than those calculated for Africans (0.438), Southeast Asians (0.395) and Europeans (0.414). These results are in line with expectations considering the ancestry shared by indigenous, American and East Asian peoples and deserve to be confirmed with additional case-control studies. On the other hand, the average genetic risk scores for obesity in indigenous and continental populations showed that mean value in indigenous (0.312) is smaller than those calculated for all continental groups that vary from 0.517 in East Asians to 0.666 in Africans and Europeans. This is certainly due to the very low frequency of the risk allele (A) of the rs8050136 (*FTO*) variant among indigenous, unlike that observed in other ethnic groups.

In summary, the epidemiological data obtained in this study reveal that the studied indigenous populations are in an initial stage of transition, which would allow health education actions to be used to contain or minimize their progression. These data are very important from an epidemiological point of view because they reflect a peculiar period in which the indigenous peoples of the Xingu had not yet been socially impacted by the construction of the Belo Monte Hydroelectric, mainly due to the abundant distribution of financial resources and subsistence items for the indigenous people, mainly from 2012. The genetic profile of indigenous people analyzed in this study was characterized by differentiation indices classified as moderate or high in relation to continental populations, but the distribution of allele frequencies among indigenous pe indicates that the majority of associations observed with T2D in continental populations can be replicated in indigenous people of the Amazon. The genetic risk scores calculated for diabetes in indigenous people are high and similar to those calculated for Americans and East Asians, while the risk scores for obesity are low, probably due to the low frequencies of the risk allele studied in the *FTO* gene. Despite the low prevalence of nutritional and metabolic alterations in most of the analyzed indigenous groups, a significant association was found between the *ADRB3* gene and abdominal obesity and excess weight, and the KCNJ11 gene with hyperglycemia for indigenous people in general. The results obtained in this pilot study also underscore the importance of knowing the frequencies of risk alleles related to complex genetic diseases in indigenous people in order to assess the potential of these variants as biological risk factors and to identify those that may constitute a panel of mutations to be used in the prevention of diseases, such as obesity and comorbidities among indigenous and admixed populations, such as those in Brazil. Moreover, the results suggest that a further analysis of the underlying genetic profile to obesity and comorbidities has good prospects to contribute important information on the genetic basis of these diseases in indigenous, especially in the context of the current epidemiological and nutritional profile of indigenous peoples in the middle Xingu after the compensation policies used for the construction of the Belo Monte Hydroelectric.
